# Causal Relationships Between Screen Use, Reading, and Brain Development in Early Adolescents

**DOI:** 10.1002/advs.202307540

**Published:** 2024-01-02

**Authors:** Mingyang Li, Ruoke Zhao, Xixi Dang, Xinyi Xu, Ruike Chen, Yiwei Chen, Yuqi Zhang, Zhiyong Zhao, Dan Wu

**Affiliations:** ^1^ Key Laboratory for Biomedical Engineering of Ministry of Education Department of Biomedical Engineering College of Biomedical Engineering & Instrument Science Zhejiang University Yuquan Campus Hangzhou 310027 China; ^2^ Department of Psychology Hangzhou Normal University Hangzhou China; ^3^ Children's Hospital Zhejiang University School of Medicine National Clinical Research Center for Child Health Hangzhou China

**Keywords:** brain development, brain volume, early adolescence, reading, screen use

## Abstract

The rise of new media has greatly changed the lifestyles, leading to increased time on these platforms and less time spent reading. This shift has particularly profound impacts on early adolescents, who are in a critical stage of brain development. Previous studies have found associations between screen use and mental health, but it remains unclear whether screen use is the direct cause of the outcomes. Here, the Adolescent Brain Cognitive Development (ABCD) dataset is utlized to examine the causal relationships between screen use and brain development. The results revealed adverse causal effects of screen use on language ability and specific behaviors in early adolescents, while reading has positive causal effects on their language ability and brain volume in the frontal and temporal regions. Interestingly, increased screen use is identified as a result, rather than a cause, of certain behaviors such as rule‐breaking and aggressive behaviors. Furthermore, the analysis uncovered an indirect influence of screen use, mediated by changes in reading habits, on brain development. These findings provide new evidence for the causal influences of screen use on brain development and highlight the importance of monitoring media use and related habit change in children.

## Graphical abstract

The causal relationships between screen use and mental health are not clear. Herein, genetic, imaging, and questionnaire data from ABCD study are used to investigate this issue in adolescents using Mendelian randomization analyses, and found causal associations between screen exposures and behavior problems and an indirect effect between screen use and brain health by the changes in reading habits.

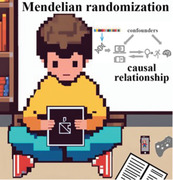

## 1 Introduction

Reading has long been considered an essential way of learning, entertainment, and brain exercise. However, rapid advances in technology have introduced a wide range of new media platforms, such as social networks, movies, and television, offering dynamic and immersive experiences that surpass traditional forms of media and transform our lifestyles. Consequently, we now dedicate more time to these platforms,^[^ ^1^, ^2^ ^]^ which may lead to a decline in reading time.^[^ ^3^, ^4^, ^5^ ^]^ Research has demonstrated that the human brain, cognition, and behavior are significantly influenced by environmental interactions, particularly during early adolescence.^[^ ^6^, ^7^ ^]^ As children nowadays spend more and more of their leisure time with new media, there is growing concern about its potential impact on brain development.^[^ ^8^, ^9^ ^]^ It is, therefore, essential to identify the potential consequences of this trend and its implications for individuals and society as a whole.

Numerous studies have shown that reading has profound benefits for brain structural and functional development. Literate individuals have greater brain volume in several regions and stronger white matter connections between the angular and dorsal occipital gyri compared to illiterate individuals.^[^ ^10^ ^]^ Moreover, literate individuals demonstrate enhanced functional activity in various brain regions during both language‐specific and general cognitive tasks.^[^ ^11^ ^]^ Even within literate individuals, reading habits have been linked to improved cognitive and behavioral performance in children,^[^ ^12^ ^]^ and reading ability is significantly correlated with brain structure and function.^[^ ^13^, ^14^ ^]^ A recent study using ABCD data found that children's reading habits contribute to their cognitive, behavioral, and brain health.^[^ ^15^ ^]^ In contrast, the relationship between screen use and child development is not yet well understood. The majority of studies indicate that prolonged screen use is associated with adverse effects on brain structure and function,^[^ ^16^, ^17^, ^18^ ^]^ as well as cognitive and behavioral problems.^[^ ^8^, ^9^, ^19^ ^]^ On the other hand, some studies also found positive effects of screen use, such as video games, on improving reading ability in dyslexic children^[^ ^20^ ^]^ and cognitive performance in normal children.^[^ ^21^, ^22^ ^]^ One possible mechanism for the negative effects of screen use is the displacement effect,^[^ ^5^ ^]^ which suggests that screen time and other activities compete for leisure time and that longer screen time may reduce other leisure activities, such as exercise and reading.^[^ ^5^, ^23^ ^]^ Regarding this hypothesis, some studies have found a moderate inverse association between screen use time and other activities,^[^ ^3^, ^4^, ^24^ ^]^ while others have reported inconsistent results.^[^ ^8^, ^23^ ^]^

However, A key missing part in those observational studies is that they mainly found associations between screen use time and certain outcomes, but not the causal effect of screen use on brain development, making it difficult to tease out the complex relationships among these variables, e.g., whether screen use triggered mental problems or vice versa or there might be a mutual interaction between the two. Causal analysis has become possible with large datasets and advanced statistical tools, such as the Mendelian randomization (MR) analysis,^[^ ^25^ ^]^ which uses genetic variation as a tool to infer causal relationships between exposure and outcome variables and has been widely used to make causal inferences in life science.^[^ ^26^, ^27^, ^28^ ^]^

**Figure 1:**
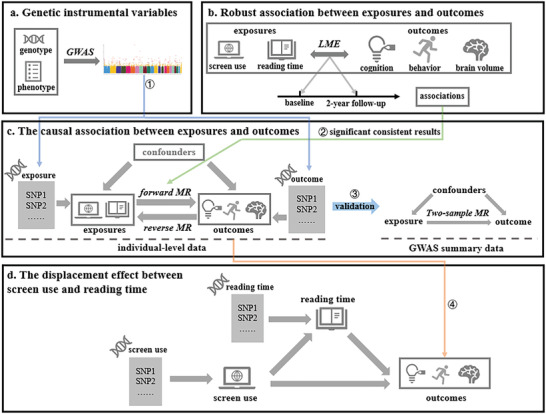
Overview of the current study. a) Genome‐wide association analysis (GWAS) was used to identify SNPs associated with phenotypes of interest, and the results would be used in further Mendelian randomization (MR) analysis (① blue line). b) Linear mixed‐effects model was used to identify robust associations between exposures (e.g., screen use and reading time) and outcomes (e.g., cognition, brain volume). Only the associations that were found to be significant at both baseline and 2‐year follow‐up were fed to the following MR analysis (② green line). c) Bidirectional one‐sample MR analysis was performed to investigate the causal relationship between exposures and outcomes, and the two‐sample MR analysis was performed to validate the causal results (③). In addition, the significant result for reading time would be used in the following analysis (④ orange line). d) Mediation and two‐step MR analysis were used to investigate the displacement effect between screen use and reading habits.

In this study, we aim to investigate the causal associations between screen use and reading habits on cognition, behavior, and brain structure in young adolescents. This study employed the longitudinal ABCD study with comprehensive imaging,^[^ ^29^ ^]^ questionnaire,^[^ ^30^, ^31^ ^]^ and biochemical measures^[^ ^32^ ^]^ from approximately 12000 participants aged between 9 and 13 years old. We first identified the specific outcome variables (such as brain volume, cognition, and behavior) that exhibit significant associations with exposure variables (such as screen use time and reading time). Subsequently, we employed one‐sample MR analysis to determine whether these associations reflect causal effects. Furthermore, we elucidated the potential displacement effect and its impact on brain development, utilizing both mediation analysis and two‐step MR analysis to test the competitive relationship between new media and reading (see **Figure** **1** for the overview of the study). We also validated the causal relationship using other cohorts by two‐sample MR analysis.

## 2 Results

### 2.1 Overview of Screen Use and Reading in Early Adolescents

**Table** **1** shows the sociodemographic characteristics of the subjects included in the baseline and 2‐year follow‐up ABCD datasets, respectively. The study investigated six screen use activities (Table 1): 1) watching television shows or movies (TVM), 2) watching videos (Video), 3) playing video games (Game), 4) texting on a cell phone, tablet, or computer (Text), 5) visiting social networking sites (SNS), and 6) video chatting (VC). At baseline, TVM was the most popular screen use activity among the participants (aged 9.87 ± 0.62 years old), with 96% of them having used it, and an average daily use of 1.21 ± 1.00 hours. This was followed by Game (86%, 0.96 ± 1.05 h) and Video (80%, 0.93 ± 1.10 h) use. Text (41%, 0.19 ± 0.42 h), VC (36%, 0.16 ± 0.38 h), and SNS (17%, 0.09 ± 0.31 h) were used less frequently. At the 2‐year follow‐up (aged 11.87 ± 0.64 years old), the daily use of all types of media had increased (Table 1). In particular, the proportion of participants who ever used Text increased from 41% to 67%, and SNS use increased from 17% to 45%. In addition, reading habits remained consistent, with 71% of children having a reading habit (averaged reading time per day > 0) at baseline (0.58 ± 0.67 h) and 77% at the 2‐year follow‐up (0.53 ± 0.44 h). It is worth noting that the baseline collection period was from 2016 to 2018, and the 2‐year follow‐up collection period was from 2018 to 2020, which partially overlapped with the COVID‐19 pandemic period, so the increase in screen use time may not only be an effect of age but may also be complicated by the epidemic. These effects were not investigated in the current study though. Moreover, we performed a correlation analysis between exposure variables by using Pearson correlation. We found a significant positive correlation among the screen exposure variables (*r* = 0.06 to 0.43, *p* < 10^−6^), and significant negative correlations between the six screen exposures and reading time (*r* = −0.15 to −0.05, *p* < 10^−4^) at baseline, which was similar at 2‐year follow‐up (Figure S1, Supporting Information).

**Table 1:** The sociodemographic, screen time, and reading time statistics of the study population.

sociodemographic characteristics	mean ± SD or percent	
	baseline (*n* = 7107)	2‐year follow‐up (*n* = 4505)
Age (years)	9.87 ± 0.62	11.87 ± 0.64
Sex		
Male	0.53	0.54
Female	0.47	0.46
Race		
White	0.661	0.688
Black	0.137	0.115
Native American	0.005	0.006
Pacific island	0.001	0.001
Asian	0.022	0.020
Others	0.173	0.170
Ethnicity		
Hispanic/Latino	0.20	0.19
Household income		
< $11999	0.28	0.05
$12 000 – $24999	0.14	0.05
$25 000 – $49999	0.23	0.13
$50 000 – $99999	0.25	0.28
> $100 000	0.10	0.49
Parent education		
college or more	0.86	0.87
Activities		
TVM	1.21 ± 1.00 (0.96)	1.45 ± 1.45 (0.90)
Video	0.93 ± 1.10 (0.80)	1.27 ± 1.57 (0.81)
Game	0.96 ± 1.05 (0.86)	1.81 ± 2.31 (0.80)
Text	0.19 ± 0.42 (0.41)	0.53 ± 0.96 (0.67)
SNS	0.09 ± 0.31 (0.17)	0.51 ± 1.10 (0.45)
VC	0.16 ± 0.38 (0.36)	0.33 ± 0.77 (0.43)
Reading	0.58 ± 0.67 (0.71)	0.53 ± 0.44 (0.77)

The values in the parentheses in the *Activities* column represent the proportion of children who used this type of activity, that is, the proportion of children whose average daily time is greater than zero in the data.

SD = standard deviation; TVM = watching television shows/movies; Video: watching videos; Game = playing video games; Text = texting on a cell phone, tablet, and so on; SNS = visiting social networking sites; VC = video chatting

### 2.2 The Causal Effect of Screen Use and Reading Habits on the Cognition and Behavior in Early Adolescents

**Figure 2:**
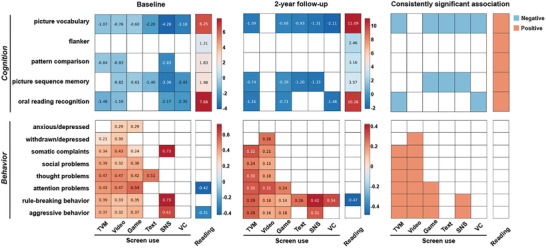
The associations between six types of screen use or reading habits and children's cognitive and behavioral performance at baseline (left) and 2‐year follow‐up (middle). The right panel shows consistent results at two time points. The colored cell indicates the significant association after Bonferroni correction, and the value indicates the coefficient (β) in the linear mixed effects model. The standard errors and significance levels corresponding to the coefficients are shown in Table S1 (Supporting Information). It is worth noting that a higher cognitive score indicates better cognitive ability, while a higher behavioral score indicates more severe behavioral problems. Abbreviation: TVM = watching television shows/movies; Video: watching videos; Game = playing video games; Text = texting on a cell phone, tablet, and so on; SNS = visiting social networking sites; VC = video chatting.

To examine the impact of different types of screen use on cognition and behavior, we used a linear mixed‐effect (LME) model to analyze the relationship between screen use and outcomes including 5 cognitive scores from the NIH cognitive toolbox and 8 behavioral outcomes from the child behavior checklist (CBCL) questionnaire at two time points. We focused on results that were significant at both time points for reliability consideration. Our results show different patterns in the cognitive and behavioral scores depending on the type of media used (**Figure** **2**; Table S1, Supporting Information). TVM use was negatively associated with two crystallized intelligence scores including picture vocabulary (*β* = −1.07 and −1.09 at two time points, *p* < 10^−6^) and oral reading recognition (*β* = −1.48 and −1.16, *p* < 10^−6^), and positively correlated with all behavioral problem scores (corrected *p* < 0.05) except for anxious/depressed and withdrawn/depressed symptoms. Video use was positively significantly associated with all behavioral problem scores (corrected *p* < 0.05) except for anxious/depressed symptoms. Game use was significantly associated with two cognition scores including picture vocabulary (*β* = −0.60 and −0.68, *p* < 10^−4^) and picture sequence memory (*β* = −0.61 and −0.39, *p* < 0.002), and three behavioral problem scores including attention problems (*β* = 0.24 and 0.39, *p* < 10^−7^), rule‐breaking behavior (*β* = 0.14 and 0.24, *p* < 10^−5^) and aggressive behavior (*β* = 0.18 and 0.27, *p* < 10^−6^). Text use was significantly associated with picture vocabulary (*β* = −0.93 and –1.56, corrected *p* < 0.004) and picture sequence memory (*β* = −1.40 and −1.20, *p* < 0.003). SNS use was significantly associated with picture vocabulary (*β* = −4.28 and −1.31, *p* < 10^−4^), picture sequence memory (*β* = −3.36 and −1.33, *p* < 10^−6^), and two behavioral problem scores including rule‐breaking behavior (*β* = 0.73 and 0.42, *p* < 10^−4^) and aggressive behavior (*β* = 0.61 and 0.31, *p* < 0.004). VC use was negatively significantly associated with picture vocabulary (*β* = −2.11 and –2.64, *p* < 10^−6^) and picture sequence memory (*β* = −1.46 and −1.90, *p* < 0.001). On the contrary, reading habits showed positive associations with all the cognition tests at two time points (*β* = 1.21 to 11.09, *p* < 0.0002), but no consistent significant associations with any of the CBCL scores (Figure 2; Table S1, Supporting Information). It is worth noting that a higher cognitive score indicated better cognitive ability, while a higher behavioral problem score indicated more severe behavioral problems. Therefore, our results confirmed that screen use was generally adversely associated with cognitive and behavioral development while reading was mostly beneficial. We also performed the Linkage Disequilibrium Score Regression (LDSC^[^ ^33^ ^]^) among these exposures to explore their intrinsic associations at the genetic level (see Supporting Results for detailed descriptions).

**Figure 3:**
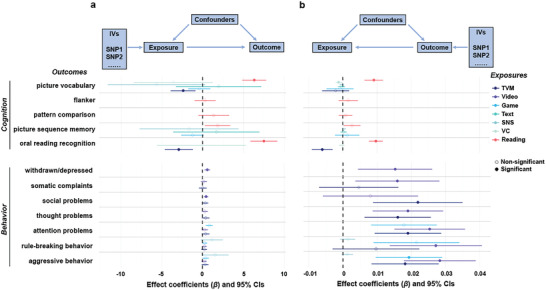
The forest plots of forward a) and reverse b) Mendelian randomization (MR) analysis between screen use or reading and children's cognition and behavior. The effect coefficients show the change in children's cognitive or behavioral scores due to daily screen time or reading per hour (forward MR), or vice versa (reverse MR), and the error bars represent 95% CIs. An effect that survived FDR correction, weak instrument test, and overidentification test was considered a significant causal effect.

Based on the above regression results, we performed MR analysis to determine the causal influence of screen use and reading habits on cognitions and behaviors in the baseline data (**Figure** **3**; Table S2, Supporting Information). We found a negative causal effect of TVM use on picture vocabulary (*β* = −2.39, corrected *p* < 0.02) and oral reading recognition (*β* = −2.93, corrected *p* < 0.007); video use on withdrawn/depressed symptoms (*β* = 0.57, corrected *p* < 0.008) and social problems (*β* = 0.43, corrected *p* < 0.02); and positive causal effect of reading on picture vocabulary (*β* = 6.31, corrected *p* < 10^−14^) and oral reading recognition (*β* = 7.50, corrected *p* < 10^−16^).

We also performed a reverse MR analysis to explore whether cognition or behavior had a causal impact on screen use or reading habit (Figure 3; Table S2, Supporting Information). The results showed a negative causal effect of picture vocabulary on VC use (*β* = −0.001, corrected *p* < 10^−8^); oral reading recognition on TVM use (*β* = −0.006, corrected *p* < 0.001); social problems on TVM use (*β* = 0.022, corrected *p* < 0.003); thought problems on TVM and Video use (*β* = 0.016 and 0.019, corrected *p* < 0.004); attention problems on TVM and Video use (*β* = 0.019 and 0.025, corrected *p* < 0.0006); rule‐breaking behaviors on Video use (*β* = 0.027, corrected *p* < 0.0006); aggressive behavior on TVM, Video, and Game use (*β* = 0.018, 0.028, and 0.019, corrected *p* < 0.001); while picture vocabulary and oral reading recognition had a positive causal effect on reading time (*β* = 0.009 and 0.010, corrected *p* < 10^−8^). The reverse causal analysis suggested that specific cognitive or behavioral problems may lead to increased screen use.

For validation of these findings, we used two‐sample MR anlaysis employing other datasets. To our knowledge, there are currently no GWAS summary data on adolescent screen use besides ABCD. Therefore, we used GWAS data on leisure screen time from UK biobank data (*n* up to 526 725^[^ ^34^ ^]^) that does not have subcategories but only the total screen use time. For cognitive outcome, we used GWAS data on reading ability (*n* = 27 180^[^ ^35^ ^]^) and general cognitive performance (*n* = 257 841^[^ ^36^ ^]^). All data were based on cohorts of European ancestry, and there was no sample overlap between screen use and reading ability but a 40% overlap between screen use and cognitive performance, which may lead to about 5% estimation bias according to the previous study.^[^ ^37^ ^]^ In the forward direction, IVW methods showed that screen use was causally associated with reduced reading ability (*β* = −0.18, *p* < 0.0004) and cognitive performance (*β* = −0.11, *p* < 0.002). Other methods, including weighted median and MR‐PRESSO, yielded similar estimates. Sensitivity analyses showed no evidence of directional pleiotropy with the MR‐Egger intercept test and no bias in the leave‐one‐out plot (Figures S2,S3, Supporting Information), and the MR‐PRESSON test showed a significant effect after correcting the horizontal pleiotropy (*β* = −0.21 and −0.15, *p* < 10^−5^).

In the opposite direction, we also observed significant causal effects of reading ability on screen use (only one instrumental SNP, *β* = −0.16, *p* < 0.03). The causal effect of cognitive performance on screen use was significant with the IVW, weighted median, and MR‐PRESSO methods (*β* = −0.25 to −0.16, *p* < 10^−7^), and there were no significant results in the MR‐Egger intercept test and no bias in the leave‐one‐out test (Figure S4, Supporting Information). The MR‐PRESSO test also showed significant estimation after correcting the horizontal pleiotropy (*β* = −0.25, *p* < 10^−14^). These results were overall consistent with the above one‐sample MR analysis that screen use has a bidirectional causal relationship with cognitive performance.

### 2.3 The Causal Effect of Screen Use and Reading Habits on the Brain Volume in the early Adolescents

**Figure 4:**
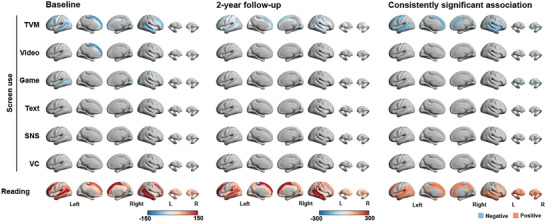
The effect coefficient maps of the associations between screen use or reading habits and children's brain volumes in 148 cortical areas and 12 subcortical areas at baseline (left) and 2‐year follow‐up (middle). The right panel shows consistently significant results at two time points. The colored brain area represents the significant association after FDR correction, and the value indicates the effect coefficients (β). The detailed results in all 148 brain regions (β, standard errors, and significance levels) corresponding to the coefficients are shown in Table S3 (Supporting Information).

We further examined the relationship between different types of screen use or reading habits and brain volumes in 160 regions, based on 3D T1‐weighted images (**Figure** **4**; Table S3, Supporting Information). The results showed that TVM use had a consistent negative association with brain volume in 32 regions (14 in the left hemisphere) at both time points. These regions were distributed in the visual, sensorimotor, prefrontal, and temporal regions. Game use was consistently negatively associated with bilateral putamen, although a wider range of associations were found at the baseline. Reading time was consistently positively associated with brain volumes in 85 regions (40 in the left hemisphere), widely distributed in the inferior parietal, temporal, and frontal regions (Figure 4).

**Figure 5:**
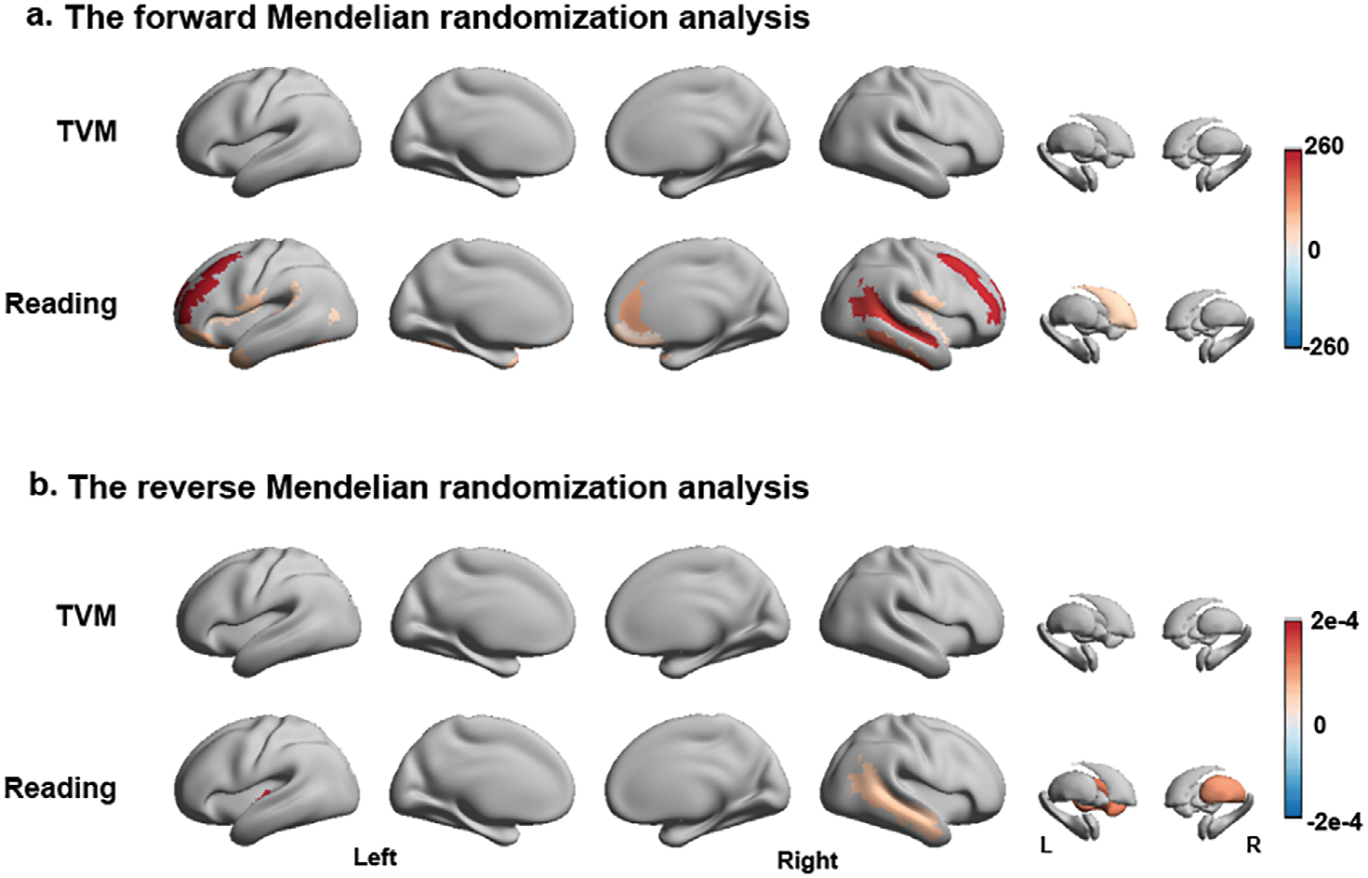
The forward a) and reverse b) Mendelian randomization (MR) analysis between screen use (i.e., TVM [watching television shows/movies]) or reading and regional brain volumes. The colored brain area represents the significant results after FDR correction, and the value indicates the change in brain volume due to daily screen time or reading per hour (forward MR), or vice versa (reverse MR). The detailed results in all brain regions (mean, standard error, and significance) are shown in Table S4 (Supporting Information). An effect that survived FDR correction, weak instrument test, and overidentification test was considered a significant causal effect.

We then applied MR analysis within the significant associations identified from the above analysis. We did not find any significant causal effect of TVM or Game use on brain volume among the 32 TVM‐related brain areas and two Game‐related brain areas. Reading habits showed significant causal influences on brain volume in 22 areas, including the bilateral prefrontal, temporal, insula regions, and left caudate (corrected *p* < 0.05, **Figure** **5**a).

In the reverse MR analysis, there is no significant causal effect of brain volume on screen use. The volumes of 5 brain regions, including the left inferior segment of the circular sulcus of the insula, the right middle temporal gyrus, the right inferior temporal sulcus, the left putamen, and the right thalamus, showed significant causal effects on reading habits, indicating better development of these brain regions may lead to higher preference in reading (β = 0.0001 – 0.0002, corrected *p* < 0.05, Figure 5b and Table S4, Supporting Information).

To validate the above results, we used two‐sample MR analysis on the GWAS data on screen use and the GWAS data on brain volume of 148 cortical regions (Destrieux atlas) from UK Biobank data (*n* = 33 224^[^ ^38^ ^]^). There was about 6% sample overlap between the two GWAS results, which can lead to less than 0.1% estimation bias according to the previous study.^[^ ^37^ ^]^ The IVW method showed no significant results between screen use and brain volume in the 148 brain regions (corrected *p* > 0.05, Table S5, Supporting Information). This result was consistent with the one‐sample MR analysis from ABCD, which found no significant causal relationship between screen use and brain volume, although the LME analysis showed associations between them.

### 2.4 Screen Use Indirectly Influences Neurocognition and Brain Volume Via the Change of Reading Habits in Early Adolescents

**Figure 6:**
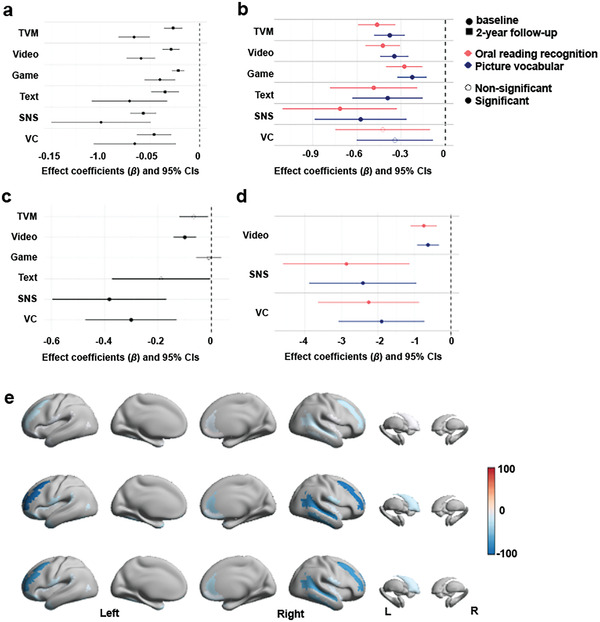
Estimation of the indirect influences of screen use on neurocognition via the change of reading habits in early adolescents. a) The associations between 6 types of screen use and daily reading time at two time points. b) The indirect effect of screen use on two reading‐related cognitions by the mediation analysis at baseline, and the result at two‐year follow‐up was similar. c) The MR analysis between 6 types of screen use and daily reading time. The effect coefficients show the change in daily reading time due to daily screen time per hour. d) The indirect effect of screen use on two reading‐related cognitions by the two‐step MR analysis. The effect coefficients show the effect of screen time on children's cognitions via changes in reading time. The error bars in the forest plot represent 95% Cis. e) The indirect effect of screen use on brain volumes of the 22 reading‐related areas by the two‐step MR analysis. The colorbar indicates the indirect effect of screen use on brain volume.

The displacement theory assumed that screen use would occupy other types of activity time, resulting in changes in corresponding outcomes. Therefore, we used reading time as an intermediate variable to test whether screen use led to a reduction in reading time, which in turn changed other outcomes like language abilities and brain volume. We first ran an LME model between screen use and reading time and found that all six types of screen use showed a significant negative association with reading time at both time points (*β* = −0.10 to −0.02, corrected *p* < 0.05, **Figure** **6**a; Table S6, Supporting Information). The MR analysis showed that the use of Video (β = −0.01, *p* < 10^−5^), SNS (β = −0.38, *p* < 0.0005), and VC (β = −0.30, *p* < 0.0006) were causally associated with reading time (Figure 6c and **Table** **2**).

**Table 2:** The causalities obtained from the Mendelian randomization analyses between screen use and daily reading time.

Screen type	*β*	*se*	*p*	Weak IV test	Sargan Test
**TVM**	−0.066	0.027	0.016	16.267	0.209
**Video**	**−0.099**	**0.022**	**0.000**	**16.477**	**0.469**
**Game**	−0.010	0.024	0.693	16.909	0.006
**Text**	−0.188	0.094	0.045	17.340	0.114
**SNS**	**−0.382**	**0.109**	**0.000**	**17.379**	**0.136**
**VC**	**−0.301**	**0.087**	**0.001**	**16.920**	**0.331**

β indicates effect coefficients from the Mendelian randomization analyses, and se is the standard error of the coefficient. An effect that survived the Bonferroni correction (corrected p < 0.05), the weak instrument test (>10), and the overidentification test (> 0.05) was considered a significant causal effect and was bolded in the table.

To investigate whether screen use indirectly influenced neurocognition through the change in reading time, we first used the mediation analysis model of traditional statistical analysis followed by the two‐step MR analysis (one kind of mediated MR method), restricting the outcome in the model to the variables that showed a significant causal relationship with reading time. In the mediation analysis, all types of screen use except VC showed a significant mediation effect on the two language abilities (corrected *p* < 0.05, Figure 6b; Table S7, Supporting Information) at both time points. Meanwhile, all 6 types of screen use showed a significant mediation effect on the brain volume in most reading‐related regions (Table S8, Supporting Information) at both time points. The two‐step MR analysis showed strong indirect effects of Video use on the two crystallized intelligence performances (picture vocabulary and oral reading recognition) related to reading time (β = −0.63 and −0.74, *p* < 0.00007, Figure 6d and Table S9, Supporting Information); moderate indirect effects of SNS and VC use on the same two cognitions (β = −2.86 to −1.90, *p* < 0.003, Table S9, Supporting Information), and weak indirect effects of those three types of screen use on brain volumes in all 22 reading‐related brain areas found in the above MR analysis (β = −96.06 to −1.48, *p* < 0.03, Figure 6e; Table S10, Supporting Information).

## 3 Discussion

The widespread use of multimedia devices in modern society is transforming our lifestyles. The genetic, imaging, and questionnaire data from the ABCD study allowed us to understand the causal relationships between screen time, reading, and various outcomes in childhood and adolescence. Our results indicate that screen use has adverse causal associations with several cognitive and behavioral outcomes, while reading has beneficial causal associations with two forms of crystallized intelligence (picture vocabulary and oral reading recognition). While the regression model shows significant associations between both reading and TVM and brain volumes in many regions, the MR analysis shows that only reading has a direct causal effect on brain volume in specific brain regions. In addition to examining the direct causal effects, we are also interested in the indirect effects of screen use on brain development via changes in reading habits. The results suggest that engagement in screen use activities, such as watching videos, leads to a reduction in time spent reading. Moreover, this decline in reading adversely impacts cognition and brain volume development that might otherwise have benefited from consistent reading practices

Previous studies have shown that screen use time has a negative impact on children's cognitive abilities, such as memory and language.^[^ ^8^, ^39^, ^40^, ^41^ ^]^ Similarly, our study found that different types of screen use were associated with lower scores on picture sequence memory, picture vocabulary, and oral reading recognition tests. The picture sequence memory test assesses visuospatial and memory skills, while the latter two tests reflect language skills and crystallized intelligence in children.^[^ ^42^ ^]^ Using MR analysis, we discovered that screen use, particularly watching TV shows and movies, had a significant causal influence on language skills, and this result was also validated in the two‐sample MR analysis. This effect may be due to the unsuitable content of TV and movies for language development, such as excessive oral expression and grammatical inaccuracies, or the passive nature of TV and movie viewing without sufficient interaction.^[^ ^41^ ^]^ Further research is needed to explore the underlying mechanisms of this influence. On the other hand, reading habits showed positive associations with all cognitive tests in the regression model. However, the MR analysis only demonstrated two language skills that were causally influenced by reading habits. These language skills are part of crystallized intelligence, which is acquired through long‐term learning.^[^ ^40^ ^]^ Other cognitive abilities related to reading, such as working memory and visual recognition, fall under fluid intelligence, which encompasses more basic cognitive abilities and is less dependent on culture and education.^[^ ^40^ ^]^ Hence, it is reasonable that the MR analysis only revealed the causal relationship between reading habits and the two language skills, while filtering out the effects on other cognitive abilities. Moreover, the inverse MR analysis revealed a significant causal effect of these two language skills on reading habits, suggesting a reciprocal relationship and a mutually enhancing cycle between reading habits and language skills during brain development.

Many studies have linked screen use to a variety of behavioral problems in children, including depression, attention problems, emotional problems, and social problems.^[^ ^8^, ^9^, ^19^, ^40^, ^43^ ^]^ Consistent with these findings, our study, using data from the CBCL questionnaires, identified associations between specific screen use activities (e.g., TV, Video, Game, and SNS) and several behavioral problems, such as withdrawal/depression, social problems, attention problems, and rule‐breaking behavior. Interestingly, we did not find a consistent and significant association between reading time and any of these behavioral problems, suggesting that behavioral problems were preferentially influenced by screen use over reading. In terms of causal relationships, our MR analyses revealed a negative causal effect between video use on withdrawn/depressed symptoms, as well as social problems. The effect of screen use on depression may be because screen use leads to reduced physical activity, sleep time, and real‐world social interaction, which are protective factors for depression symptoms. The violent scenes and rude ways of socializing on TV or video shows might contribute to the finding that screen use causes social problems in young adolescents. Therefore, to improve the healthy development of children in the digital age, scientific instruction like the Canadian 24‐Hour movement guidelines (https://csepguidelines.ca/) should be proposed to regulate children's use of new media, as well as to ensure children's time in other activities such as reading, physical activity, and sleep.^[^ ^44^ ^]^

Interestingly, the reverse MR analysis revealed that other behavioral problems, such as attention problems, rule‐breaking behavior, and aggressive behavior, may be causes rather than consequences of screen use, indicating a complex interaction between cognitive behavior and screen use. Therefore, while caution is still warranted regarding screen use in children, it's essential not to overstate the negative effects and consider the complex interplay between screen use and behavioral problems. This finding was not further verified with two‐sample MR because we did not find existing GWAS data for related behavioral phenotypes.

The associations between screen time and alterations in brain structure have been consistently reported in prevoius studies,^[^ ^17^, ^18^, ^45^, ^46^ ^]^ but there was no evidence of whether these relationships are causal. We found a similar negative effect of screen use on brain volume to the previous research.^[^ ^17^ ^]^ However, the MR analysis showed there was no causal influence of screen use on brain volume in both one‐sample and two‐sample MR analyses. This suggests that the observed negative correlation between screen use and brain volume could be attributed to other unobserved factors in previous related studies. For example, boys, lower income, and lower parental education were associated with higher total screen use time, while girls, higher income, and higher parental education were associated with higher reading time, suggesting the demographic and socioeconomic statistics on the children's habits based on our statistics. Therefore, we took these variables as covariates in our analysis to reduce estimation bias. However, brain volume is not the most sensitive measure, and previous studies have found that screen use affects brain function,^[^ ^16^, ^47^ ^]^ but whether functional changes are before structural changes (or vice versa), does not seem to have been studied, which is an interesting question for further research. Therefore, future studies could incorporate multimodal neuroimage data to explore the causal effects of screen use on specific changes in brain function or the influence of microstructure in both gray and white matter areas.

In contrast, reading habits were positively associated with brain volume in multiple areas, including bilateral prefrontal, insula, and temporal lobes without obvious laterality. The prefrontal cortex is essential for higher‐level functions such as cognitive control and executive function.^[^ ^48^ ^]^ Insula has been associated with neuropsychiatric disorders such as anxiety and depression, as well as language abilities like speech production.^[^ ^49^ ^]^ The temporal lobe is an important association cortex that plays an important role in language comprehension and integration.^[^ ^13^ ^]^ Reading habits may improve their language skills by modulating the structural properties of these brain regions. These findings were consistent with a recent study on reading time using ABCD data,^[^ ^15^ ^]^ despite the different analysis approaches. Meantime, our findings align with earlier findings of larger brain volumes in literate individuals compared to illiterate individuals.^[^ ^10^ ^]^ A number of other studies have also demonstrated correlations between reading ability and brain volume.^[^ ^50^, ^51^, ^52^ ^]^

In addition to the direct effects of screen time on brain development, we also examined the displacement effect between screen use and reading. Previous research has found that new and old media compete for leisure time^[^ ^4^, ^5^ ^]^ and that the two may have opposite effects on brain health.^[^ ^3^ ^]^ Our study further revealed that increased engagement in activities such as Video and SNS may result in reduced reading habits, thereby diminishing the cognitive and brain volume benefits from reading in children. For instance, although screen use did not directly alter brain volume, it showed an indirect adverse effect on brain structure via the change in reading. Overall, our findings provide partial support for the displacement hypothesis,^[^ ^5^, ^24^ ^]^ suggesting that new media activities can displace reading habits in children and adolescents. Future studies encompassing a broader range of activities are needed to investigate the influence of new media on the next generation.

## 4 Limitation

Although ABCD study has a large sample size and a wealth of scale, the participants were dominated by white people, lacking the representation of other races. Besides, the sample size in ABCD is still small for GWAS analysis, resulting in limited statistical power to detect the effective variant. Our study solely relied on cognitive scores from the NIH Cognition Toolbox, which may not encompass skills such as hand‐eye coordination or reaction time that could potentially be improved through activities like video gaming. Including a broader range of cognitive and behavioral measures in future studies would offer a more comprehensive understanding of the developmental benefits and drawbacks associated with screen use. Furthermore, our correlation analysis was limited to two time points and the second time point was used for consistency check. The screen use patterns are dynamic and may continually change with age, and the effects of media exposure on children may accumulate over time. Therefore, the inconsistent results observed between the two time points may hold meaningful insights, which we did not account for in our study. Moreover, although MR analysis uses genetic information to estimate the causal influence of exposures on outcomes, the instrumental variables (SNPs) might still be associated with outcomes through potential confounders rather than direct influences.^[^ ^25^ ^]^ Meantime, the causal effects were estimated by the genetic variation rather than a randomized controlled trial, so the results need to be interpreted with caution.^[^ ^53^ ^]^ Lastly, it is important to note that the results of an MR analysis can be influenced by sample selection biases.^[^ ^54^ ^]^ Therefore, we tested some findings using two‐sample MR analyses, but these data were from older cohorts (such as the UK Biobank) rather than adolescents, thus introducing age bias, and the lack of cognitive or behavioral specificity in the phenotypes also hampered our understanding of the effect of screen use. Meantime, due to the lack of GWAS data for reading habits in other cohorts, two‐sample MR on the mediated effect of reading could not be performed. Thereby, large cohort studies in the children and adolescent population are desired to enable a rigorous genetic analysis in feature research.

## 5 Conclusion

Our study employed MR analysis to investigate the causal impact of screen use and reading on brain development in early adolescents. The results indicate that screen use has both direct and indirect effects on brain development. On the other hand, it is important to avoid exaggerating the negative consequences of screen use, as some behavioral problems may precede and contribute to excessive screen use. In summary, these findings underscore the importance of monitoring media use and the associated changes in habits resulting from media exposure. Taking proactive measures to promote the healthy development of young minds in the digital age is crucial.

## 6 Experimental Section

To examine the relationship between exposure variables (e.g., reading and six screen exposures) and cognitive skills, behavior, and brain volume. A LME model was first used to explore the association between these variables. To infer a causal relationship between the variables, a one‐sample MR analysis based on the LME analysis results was performed. Considering the small sample size of ABCD for GWAS analysis, the results of one‐sample MR analysis were further validated by two‐sample MR analysis using larger sample datasets. To examine whether screen use indirectly influences the outcomes by changing reading habits (namely, the displacement effect), a mediation analysis was first conducted with reading time as the mediator and then performed a two‐step MR analysis. Detailed descriptions of the methods used in this study can be found in the Supporting Information.

## Conflict of Interest

The authors declare no conflict of interest.

## Supporting information

Supporting Information

Supplemental Tables 1‐14

## Data Availability

The data that support the findings of this study are available from the National Institute of Mental Health Data Archive (NDA). Restrictions apply to the availability of these data, which were used under license for this study. Data are available at https://nda.nih.gov/abcd with the permission of NDA institution.
