# Supplementary material for: Causal Relationships Between Screen Use, Reading, and Brain Development in Early Adolescents
Source: Adv Sci (Weinh). 2024 Jan 2;11(11):2307540. doi: 10.1002/advs.202307540 (PMC10953555; doi:10.1002/advs.202307540)
Supplement: Supplementary file 1 — Supporting Information [file ADVS-11-2307540-s002.pdf]

ADVANCED SCIENCE
Open Access

Supporting Information

for *Adv. Sci.*, DOI 10.1002/advs.202307540

# Causal Relationships Between Screen Use, Reading, and Brain Development in Early Adolescents

*Mingyang Li, Ruoke Zhao, Xixi Dang, Xinyi Xu, Ruike Chen, Yiwei Chen, Yuqi Zhang, Zhiyong Zhao and Dan Wu**

# Supporting Information

**Causal Relationships Between Screen Use, Reading, and Brain Development in Early Adolescents**

*Mingyang Li, Ruoke Zhao, Xixi Dang, Xinyi Xu, Ruike Chen, Yiwei Chen, Yuqi Zhang, Zhiyong Zhao, Dan Wu**

## Supporting Results

### Linkage Disequilibrium Score Regression analysis

We performed the Linkage Disequilibrium Score Regression (LDSC [1]) analysis among the exposures to explore the genetic associations among them. However, the relatively small sample size in the current dataset is not well powered to detect the heritability, which affected the estimate of genetic correlation [2]. The VC showed negative heritability estimates ($h^2$ = -0.047, *se* = 0.071, $p > 0.2$, **Table S11**) and this might result from random noise or misspecification of the standard additive mechanism [3], so we removed it from the following LDSC analysis. Among the rest exposures, positive genetic correlations were observed among the screen exposure variables ($r_g$ = 0.29 to 5.36) except for the correlation between video and TVM ($r_g$ = -0.19), SNS and Video ($r_g$ = -7.70), SNS and Game ($r_g$ = -5.36). Negative genetic correlations were found between the five screen exposures and reading time ($r_g$ = -2.53 to -0.07). However, only the genetic correlation between TVM and reading time was significant

($r_g$ = -1.34, $p < 0.0002$, **Table S12**). The absolute value of genetic correlation beyond one is due to the small heritabilities in the exposure variables (TVM, $h^2$ = 0.103; SNS $h^2$ = 0.0018; **Table S11**) given that the $r_g$ is multiplied by the reciprocal of the square root of the $h^2$ of the two variables [1].

Next, we calculated the genetic correlations between the exposures and outcomes based on the significant results in the regression analyses. For the non-imaging outcomes, there were 22 calculable pairs and the direction of most genetic correlations was the same as the direction of statistical regression (**Table S13**), but only the correlation between reading time and two language abilities (picture vocabulary: $r_g$ = 1.25, FDR corrected p < 0.003; oral reading recognition: $r_g$ = 1.38; FDR corrected $p < 0.002$) were significant. For imaging outcomes, TVM or Game showed generally negative genetic correlations to the brain volume in most brain regions while reading time showed generally positive genetic correlations, but none of those results survived the FDR correction (**Table S14**). In summary, the genetic correlation showed a similar trend to the statistical regression, but the small sample size for genetic analysis may not be sufficient to detect the significant heritabilities and intrinsical correlations for those exposures.

## Supporting Methods

### Participants

This study utilized data from the Adolescent Brain Cognitive Development (ABCD) study, version 4.0. The ABCD study is a large-scale longitudinal tracking study that aims to investigate

brain development in children and adolescents. The dataset utilized in this study included brain imaging data, questionnaires, and genetic data collected during both the baseline and two-year follow-up assessments. The ABCD study recruited 11,875 children aged 9 and 13 from 21 research sites across the United States between October 2016 and October 2018. The same cohort was reassessed using similar measurements two years later. All study procedures were approved by institutional review boards at individual sites, and written consent was obtained from parents with verbal assent from children. Comprehensive details about the ABCD study such as data types, preprocessing, and ethical considerations can be found elsewhere (Developmental Cognitive Neuroscience Special Issue 2018, vol. 32, pp. 1–164). We excluded preterm-born infants with gestational age less than 37 weeks (n = 1629) from our analyses. For siblings, we randomly excluded one participant from each pair with a closed gene relationship (pi-hat > 0.4) estimated with individual genotype data using PLINK (v1.9). Finally, 7107 subjects remained at baseline and 4505 at two-year follow-up (see Table 1 for detailed sociodemographic information). Note that the reduction in subjects at the 2-year follow-up is mainly because ABCD Release 4 did not fully open the second year's data. Meanwhile, the removed siblings were identical at both time points.

**Exposure variables**

The study focused on analyzing six different exposure variables related to screen use and reading time. Screen use time was obtained from the ABCD Youth Screen Time Survey [4,5]. This survey involved self-reporting by the children regarding their daily time spent on various electronic activities including watching TV shows or movies (TVM), watching videos (Video),

playing video games (Game), texting on a device (Text), visiting social networking sites (SNS), and video chatting (VC) on both weekdays and weekends. A weighted mean time was calculated using the formula [(5*$T_{weekday}$ + 2*$T_{weekend}$)/7] to represent the daily time for each screen type. At the 2-year follow-up, the survey was slightly modified, and the time spent playing video games was split into single-player and multiplayer [6]. Therefore, we summed these two times together to get the daily time for video games.

Reading time was assessed using the ABCD Parent Sports and Activities Involvement Questionnaire (SAIQ), which was a parent-reporting questionnaire for information about the involvement in sports, music, reading, and other hobbies for the children [5]. The weekly reading time reported by the parents was divided by 7 to calculate the average reading time per day. For the 2-year follow-up, the longitudinal version of the SAIQ was used. Because children's self-reported reading time was not collected in the first two years of the ABCD study, we used the parent-reporting questionnaire. Nevertheless, all the assessments in the ABCD study were well-designed and proven to be reliable and validated by other studies [7,8]. To further ensure the reliability of our results, we used the data from two time points for cross-validation, and only the consistently significant results would enter the subsequent analysis, which may reduce the bias due to the inaccuracies in the questionnaires.

**Non-neuroimage outcome variables**

Non-neuroimaging variables in this study included children's cognitive ability and behavior. Cognitive ability was assessed using five age-corrected standard scores obtained from the NIH Toolbox [5,9], including picture vocabulary, flanker, pattern comparison processing speed,

picture sequence memory, and oral reading recognition tests. Two other tests (list sorting working memory test and dimensional change card sort) were not available during the second assessment and were therefore excluded from the analysis. Behavior was measured according to the ABCD Parent-Child Behavior Checklist Scores Aseba (CBCL), which assesses 8 behavioral dimensions (t-scores), including anxious/depressed symptoms, withdrawn/depressed symptoms, somatic complaints, social problems, thought problems, attention problems, rule-breaking behavior, and aggressive behavior [5,10].

**Neuroimaging variables**

The acquisition and preprocessing of image data have been described in detail in other articles [11,12]. Briefly, MRI scans were acquired using 3T scanners at all participating ABCD sites, with optimized and standardized acquisition protocols. A unified pipeline [11] was then employed for data preprocessing. The preprocessing of T1-weighted and T2-weighted images involved the correction of gradient nonlinearity and intensity inhomogeneity, rigid registration to a reference brain in standard space, and segmentation of brain tissues. Quality control measures were implemented both before and after the image processing to ensure the exclusion of poor-quality images. This study focused specifically on brain volume estimated using the Destrieux atlas (148 ROIs in the cortex [13]) in combination with the subcortical regions in the ASEG atlas [14] (12 subcortical regions including thalamus, caudate, putamen, pallidum, hippocampus, and amygdala in both hemispheres).

**Confounding variables**

To reduce unbiased results, we adjusted for common confounding variables in our study, including age, sex, household income, parental education, race, ethnicity, prematurity, sibling, and site effect. Race was categorized into six groups: white, black, Native American, Pacific Islander, Asian, and other. For the sake of brevity, Table 1 shows only the household income of the five levels (< $11999, $12000 - $24999, $25000 - $49999, $50000 - $99999, > $100000 annually) and the proportion of parents with a college education. In addition, because there were multiple MRI machines at the same site, we used the serial number of the MRI scanner (to take into account differences MRI scanners at the same site) as a covariate in the analysis of brain volume. For simplicity, we also referred to this as the "site effect".

**Genotype data and processing**

In our study, we used the public release 3.0 genotype data in the ABCD (v4) datasets. The dataset included both pre- and post-imputed genotype data. We referred to the previous methods in the related paper [15] to estimate population stratification, and combined the pre-imputed genotype data with the 1000 Genome Phase 3 data (https://www.internationalgenome.org/). We used the first five principal components to identify population clusters using UMAP, resulting in 7 broad populations including Africans, Americans, Bengali, Finnish Europeans, Non-Finnish Europeans, East Asians, and South Asians. Only non-Finnish Europeans ($n$ = 6161) were included in the subsequent gene-based analyses in the ABCD data.

We performed post-imputation quality control to eliminate SNPs with low minor allele frequencies (MAF < 0.01), poor imputation ($r^2$ < 0.3), high missingness (> 0.05), and those that

failed the Hardy-Weinberg disequilibrium test ($p$ < 1e$^{-6}$). Finally, 11,540,083 autosomal SNPs were included in the subsequent GWAS analysis.

We performed the GWAS for phenotypic variables (e.g., exposure and outcome variables) using the fastGWA method [16] implemented in Genome-wide Complex Trait Analysis (GCTA [17]). We included age, sex, and the top 20 principal components as covariates and accounted for relatedness using a sparse genetic relatedness matrix (GRM) with a cutoff of 0.05. The GWAS results of screen use and reading will be used for subsequent Mendelian randomization (MR) analysis.

In this study, we used genetic data in a public dataset to obtain potential causal relationships between variables. However, the use of genetic data also carries certain risks, including invasion of privacy, genomic discrimination, and other ethical and legal issues [18]. Therefore, before using genetic data, it is best to fully understand the relevant policies and regulations and comply with the data use norms.

**Statistical analyses**

To examine the relationship between exposure variables (e.g., reading and screen use) and cognitive skills, behavior, and brain volume. We first used a linear mixed-effects (LME) model to explore the association between these variables. To infer a causal relationship between the variables, we performed a one-sample MR analysis based on the LME analysis results. Considering the small sample size of ABCD for GWAS analysis, the results of one-sample MR analysis might be affected by sample selection bias [19]. We attempted to use related GWAS summary data from larger sample cohorts to perform two-sample MR analysis, to validate the

results of one-sample MR. It is important to note that for some of the variables we used, GWAS summary data were not available, and the existing data were based on adults rather than adolescents. To examine whether screen use indirectly influences the outcomes by changing reading habits, we first conducted a mediation analysis with reading time as the mediator and then performed a two-step MR analysis. In all statistical analyses, we only included participants with complete data to ensure data quality.

## Association analysis

LME models were used to examine the association between each exposure variable and each outcome variable (e.g. 5 cognitive scores, 8 behavioral scores, and brain volume in 160 brain regions). Each exposure and outcome variable pair was analyzed separately, while fixed effects for age, sex, race, ethnicity, household income, parental education, and random effects for the site were included in all models. The *fitlme* function in MATLAB (v2018a) was used for model fitting.

$$outcome \sim (1|\mathrm{site}) + age + sex + ethnicity + education + income + exposure + \varepsilon$$

Association analysis was performed separately at two time points. Bonferroni correction for multiple comparisons was applied at each time point for all non-neuroimage outcome variables, and FDR correction was applied for neuroimage variables, because conservative Bonferroni correction for hundreds of brain regions may be too strict considering that we only included results that were significant at both time points.

## Genetic correlation

We estimated the genetic correlation among seven exposure variables and their genetic correlation to the outcomes. The genetic correlations were estimated by the LDSC [1] method implemented in the Genomic SEM [20] package in the R software.

**One Sample Mendelian randomization analysis**

To examine the causal relationship between exposure and outcome variables, we used the one-sample MR method [21] in this study. This method involves two-stage least-squares (TSLS) regression with SNPs as instrumental variables (IVs) [22], we used the *OneSampleMR* R package to perform the analysis and used a liberal threshold of $p < 1e^{-5}$ and MAF > 0.005 in selecting the instruments for the exposure variables to maximize the power of the genetic instruments [22]. Linkage disequilibrium (LD) pruning with an $R^2$ threshold of 0.001 and a window size of 1 Mb to remove SNPs in high LD. SNPs that showed significant correlations with the outcome variable (using the same *p*-value threshold as for the exposure variable) were removed. Each SNP was coded as 0, 1, or 2, depending on the number of effect alleles carried by a subject. We included covariates such as age, sex, household income, parental education, and site in both regressions. The ABCD baseline data were used for the analysis because the genetic data is only available at baseline.

The MR analysis was conducted among the significant results in the above association analysis, and the FDR correction was used for multiple comparisons in all non-neuroimage ($n = 33$) and neuroimage ($n = 119$) outcome variables, respectively. Furthermore, the reverse MR analysis was also conducted to investigate the causal influence of those outcomes on the children's

screen use and reading habits. The reverse MR analysis was similar to the above MR method except that we exchanged the exposure and outcome variables [23].

The sensitivity analysis includes weak instruments and the Sargan test. In the one-sample MR analysis, if the SNPs-exposure associations were weak, chance variation means that genetic association with the exposure and outcome are correlated in the direction of the confounded association between the two, resulting in increased false positive rates [24]. Weak instrument test is performed using the F statistic for one-sample MR and the F should be greater than 10 [25]. In addition, the Sargan test is applicable if the number of instrumental variables exceeds the number of endogenous variables, and a positive test result means that at least one instrumental variable is not valid. Therefore, significant results were needed to meet three criteria: original p-value survived after FDR correction, $F > 10$ in the weak instruments test, and not significant in the Sargan test.

**Two-sample Mendelian randomization analysis**

For screen use, we did not find GWAS summary data for the above six types of screen use, so we used GWAS data on leisure screen time using UK Biobank data (*n* up to 526725 [26]). For cognitive ability, we used GWAS data on reading ability (*n* = 27180 [27]) and general cognitive performance (*n* = 257841, [28]). We selected the instrumental SNPs using the PLINK software and took the genomic data of the European superpopulation in the 1000 Genomes Project as the LD reference, which was restricted to bi-allelic SNPs with minor allele frequency > 0.1. We set the LD pruning $r^2 = 0.001$, the window size = 10000 kilobase pairs, and the P value associated with the exposures of $5e^{-8}$. We then removed the SNPs that showed significant

association with outcomes ($p$ < 5e$^{-8}$) and harmonized the data using the *harmonise_data* function in the TwoSmpleMR package to ensure that the same allele was used to estimate the genetic variant association. Inverse variance-weighted (IVW) regression [29] with multiplicative random effects was conducted as the primary analysis method. For the significant results with the IVW method, we would conduct four other methods to assess the robustness of the results including weighted median method, weighted mode, MR-Egger, and MR-PRESSO. The Wald ratio method would be used when only one instrument was available. All of these methods were conducted in the TwoSmpleMR package. In addition, we performed the MR-Egger regression to examine the potential bias of directional pleiotropy [30], the MR-PRESSO method to the horizontal pleiotropy [31], and a leave-one-out analysis to check whether the causal association was driven by a single SNP. There was no overlap between the data used in the one-sample and two-sample MR analyses.

Both one- and two-sample MR use SNPs as instrumental variables to estimate the causal effects of the exposure on the outcome, and both methods need to satisfy three assumptions, namely relevance assumption, independence assumption, and exclusion restriction. However, one-sample MR uses individual-level data and is usually estimated by the TSLS method, while two-sample MR generally uses GWAS summary data from two samples and is usually estimated by the IVW method [23,25]. Given the difference in these basic settings, there are both advantages and disadvantages for the two MR methods. On the one hand, the same population in the one-sample MR could guarantee the homogeneity in the association of the genetic variants with the exposure and outcomes, while the different samples in the two-sample MR may affect the validity of the instrumental variable assumptions because a genetic

variant may not be as strongly associated with the exposure in the outcome dataset. Meantime, the difference in population characteristics such as age, sex, and socio-economic background might affect the interpretation of causal estimates in the two-sample MR analysis. One-sample MR does not suffer from these problems, and it is even possible to compare the results of traditional statistical and MR analyses in the same study [24]. On the other hand, the overlap of participants will introduce sample selection biases and inflated false positive rate (type 1 error) in the one-sample MR analysis [19,32], while two-sample MR without sample overlap could avoid such bias [24]. Therefore, we combined the analyses with the two methods to enhance the reliability of the conclusion in the present study.

**Mediation analysis**

To estimate the mediating effect of reading habits on the relationship between screen use and outcome variables, we conducted a mediation analysis using the mediation toolbox (https://github.com/canlab/MediationToolbox) in MATLAB v2018a. Specifically, the six types of screen use were the exposure variables, reading time was the mediator variable, those that showed a significant causal relationship with reading in the above analyses were taken as the outcome variables in the mediation analysis, and the covariates including age, gender, ethnicity, race, household income, parental education, and site were also considered in the analysis. Mediation analysis was conducted at baseline and two-year follow-up, respectively.

**Two-step Mendelian randomization analysis**

Two methods could be used to estimate the mediator effect in an MR framework, namely two-step MR analysis and multivariable MR analysis. The two-step MR analysis estimates the effect of the exposure on the outcome and compares this to the product of the effect of the exposure on the mediator multiplied by the effect of the mediator on the outcome. The multivariable MR analysis compares the standard model containing only the exposure variable and the model including the mediator and the exposure, to reflect the direct and indirect effect of the exposure on the outcome [24].

To validate the above mediation analysis and generate a potential causal reference about the displacement relationship between screen use and reading habits in this study, we conducted a two-step MR analysis [23] to estimate the mediated effect of reading habits on the relationship between screen use and outcome variables. In the first step, we used MR analysis to reveal the causal effect of screen use on reading habits. In the second step, we used MR analysis to estimate the causal effect of the mediator (i.e., reading) on the outcome variables, which are significantly influenced by the mediator from the above analysis. We then used the "product of coefficients" method [33] to estimate the indirect effect and the "delta" method [34] to estimate the confidence interval [22]. Particularly, the indirect coefficient was equal to the product of two estimations (*a * b*, where *a* and *b* were the effect estimations in the above two steps, respectively), and the standard error in calculating the confidence interval was estimated as follows:

$$SE_{(\hat{a}\hat{b})} = \sqrt{(aSE(\hat{b}))^2 + (bSE(\hat{a}))^2 + SE(\hat{a})^2 SE(\hat{b})^2}$$

**Reference**

[1] B. Bulik-Sullivan, H. K. Finucane, V. Anttila, A. Gusev, F. R. Day, P. R. Loh, L. Duncan, J. R. B. Perry, N. Patterson, E. B. Robinson, M. J. Daly, A. L. Price, B. M. Neale, Nat. Genet. 2015, 47, 1236.

[2] A. Carrión-Castillo, P. M. Paz-Alonso, M. Carreiras, Nat. Hum. Behav. 2023, 51, 1120.

[3] D. Steinsaltz, A. Dahl, K. W. Wachter, Genetics 2020, 215, 343.

[4] J. M. Nagata, K. T. Ganson, P. Iyer, J. Chu, F. C. Baker, K. P. Gabriel, A. K. Garber, S. B. Murray, K. Bibbins-domingo, J. Pediatr. 2021, 240, 213.

[5] D. M. Barch, M. D. Albaugh, S. Avenevoli, L. Chang, D. B. Clark, M. D. Glantz, J. J. Hudziak, T. L. Jernigan, S. F. Tapert, D. Yurgelun-todd, N. Alia-klein, A. S. Potter, M. P. Paulus, D. Prouty, R. A. Zucker, K. J. Sher, Dev. Cogn. Neurosci. 2018, 32, 55.

[6] J. M. Nagata, G. Singh, O. M. Sajjad, K. T. Ganson, A. Testa, D. B. Jackson, S. Assari, S. B. Murray, K. Bibbins-domingo, F. C. Baker, Pediatr. Res. 2022, 92, 1443.

[7] T. L. Jernigan, S. A. Brown, Dev. Cogn. Neurosci. 2018, 32, 1.

[8] N. R. Karcher, D. M. Barch, Neuropsychopharmacology 2020, 46, 131.

[9] M. Luciana, J. M. Bjork, B. J. Nagel, D. M. Barch, R. Gonzalez, S. J. Nixon, M. T. Banich, Dev. Cogn. Neurosci. 2018, 32, 67.

[10] M. Wainberg, G. R. Jacobs, A. N. Voineskos, Mol. Psychiatry 2022, 27, 2731.

[11] D. J. Hagler, S. Hatton, M. D. Cornejo, C. Makowski, D. A. Fair, A. Steven, M. T. Sutherland, B. J. Casey, D. M. Barch, M. P. Harms, R. Watts, J. M. Bjork, H. P. Garavan, L. Hilmer, C. J. Pung, C. S. Sicat, J. Kuperman, H. Bartsch, F. Xue, M. M. Heitzeg, A. R. Laird, T. T. Trinh, R. Gonzalez, S. F. Tapert, M. C. Riedel, L. M. Squeglia, L. W. Hyde, M. D. Rosenberg, E. A. Earl, K. D. Howlett, F. C. Baker, M. Soules, J. Diaz, O. Ruiz, D. Leon, W. K. Thompson, M. C. Neale, M. Herting, E. R. Sowell, R. P. Alvarez, S. W. Hawes, M. Sanchez, J. Bodurka, F. J. Breslin, A. Shef, M. P. Paulus, W. K. Simmons, J. R. Polimeni, A. Van Der Kouwe, A. S. Nencka, K. M. Gray, C. Pierpaoli, J. A. Matochik, A. Noronha, W. M. Aklin, K. Conway, M. Glantz, E. Hoffman, R. Little, M. Lopez, V.

Pariyadath, S. Rb, D. L. Wolff-hughes, R. Delcarmen-wiggins, S. W. Feldstein, O. Miranda-dominguez, B. J. Nagel, A. J. Perrone, D. T. Sturgeon, A. Goldstone, A. Pfefferbaum, K. M. Pohl, D. Prouty, K. Uban, S. Y. Bookheimer, M. Dapretto, A. Galvan, K. Bagot, J. Giedd, M. A. Infante, J. Jacobus, K. Patrick, P. D. Shilling, R. Desikan, Y. Li, L. Sugrue, M. T. Banich, N. Friedman, J. K. Hewitt, C. Hopfer, J. Sakai, J. Tanabe, L. B. Cottler, S. Jo, L. Chang, C. Cloak, T. Ernst, G. Reeves, D. N. Kennedy, S. Heeringa, S. Peltier, J. Schulenberg, C. Sripada, R. A. Zucker, W. G. Iacono, M. Luciana, F. J. Calabro, D. B. Clark, D. A. Lewis, B. Luna, C. Schirda, J. J. Foxe, E. G. Freedman, D. W. Mruzek, M. J. Mason, R. Huber, E. Mcglade, A. Prescot, P. F. Renshaw, D. A. Yurgelun-todd, N. A. Allgaier, J. A. Dumas, M. Ivanova, A. Potter, P. Florsheim, C. Larson, K. Lisdahl, M. E. Charness, B. Fuemmeler, J. M. Hettema, H. H. Maes, J. Steinberg, A. P. Anokhin, P. Glaser, A. C. Heath, P. A. Madden, A. Baskin-sommers, R. T. Constable, S. J. Grant, G. J. Dowling, S. A. Brown, T. L. Jernigan, A. M. Dale, Neuroimage 2019, 202, 116091.

[12] B. J. Casey, T. Cannonier, M. I. Conley, A. O. Cohen, D. M. Barch, M. M. Heitzeg, M. E. Soules, T. Teslovich, D. V Dellarco, H. Garavan, C. A. Orr, T. D. Wager, M. T. Banich, N. K. Speer, M. T. Sutherland, M. C. Riedel, A. S. Dick, J. M. Bjork, K. M. Thomas, B. Chaarani, M. H. Mejia, D. J. Hagler, M. D. Cornejo, C. S. Sicat, M. P. Harms, N. U. F. Dosenbach, M. Rosenberg, E. Earl, H. Bartsch, R. Watts, J. R. Polimeni, J. M. Kuperman, D. A. Fair, A. M. Dale, Dev. Cogn. Neurosci. 2018, 32, 43.

[13] C. Destrieux, B. Fischl, A. Dale, E. Halgren, Neuroimage 2010, 53, 1.

[14] B. Fischl, D. H. Salat, E. Busa, M. Albert, M. Dieterich, C. Haselgrove, A. Van Der Kouwe, R. Killiany, D. Kennedy, S. Klaveness, A. Montillo, N. Makris, B. Rosen, A. M. Dale, Neuron 2002, 33, 341.

[15] V. Warrier, A. S. F. Kwong, M. Luo, S. Dalvie, J. Croft, H. M. Sallis, J. Baldwin, M. R. Munafò, C. M. Nievergelt, A. J. Grant, S. Burgess, T. M. Moore, R. Barzilay, A. Mcintosh, M. H. Van Ijzendoorn, C. A. M. Cecil, Lancet Psychiatry 2020, 8, 373.

[16] L. Jiang, Z. Zheng, T. Qi, K. E. Kemper, N. R. Wray, P. M. Visscher, J. Yang, Nat. Genet. 2019, 51, 1749.

[17] J. Yang, S. H. Lee, M. E. Goddard, P. M. Visscher, Am. J. Hum. Genet. 2011, 88, 76.

[18] M. C. de Hemptinne, D. Posthuma, Nat. Neurosci. 2023, 26, 932.

[19] R. A. Hughes, N. M. Davies, D. Smith, K. Tilling, Epidemiology 2019, 30, 350.

[20] A. D. Grotzinger, M. Rhemtulla, R. de Vlaming, S. J. Ritchie, T. T. Mallard, W. D. Hill, H. F. Ip, R. E. Marioni, A. M. McIntosh, I. J. Deary, P. D. Koellinger, K. P. Harden, M. G. Nivard, E. M. Tucker-Drob, Nat. Hum. Behav. 2019, 3, 513.

[21] T. M. Palmer, J. A. C. Sterne, R. M. Harbord, D. A. Lawlor, N. A. Sheehan, S. Meng, R. Granell, G. D. Smith, V. Didelez, Am. J. Epidemiol. 2011, 173, 1392.

[22] X. Liu, X. Tong, Y. Zou, X. Lin, H. Zhao, L. Tian, Z. Jie, Q. Wang, Z. Zhang, H. Lu, L. Xiao, X. Qiu, J. Zi, R. Wang, X. Xu, Nat. Genet. 2022, 54, 52.

[23] E. Sanderson, M. M. Glymour, M. V. Holmes, H. Kang, J. Morrison, M. R. Munafò, T. Palmer, C. M. Schooling, C. Wallace, Q. Zhao, G. Davey Smith, Nat. Rev. Methods Prim. 2022, 2, 6.

[24] S. Burgess, G. Davey Smith, N. M. Davies, F. Dudbridge, D. Gill, M. M. Glymour, F. P. Hartwig, Z. Kutalik, M. V. Holmes, C. Minelli, J. V. Morrison, W. Pan, C. L. Relton, E. Theodoratou, Wellcome Open Res. 2023, 4, 186.

[25] N. M. Davies, M. V. Holmes, G. Davey Smith, BMJ 2018, 362, k601.

[26] Z. Wang, A. Emmerich, N. J. Pillon, T. Moore, D. Hemerich, M. C. Cornelis, E. Mazzaferro, S. Broos, T. S. Ahluwalia, T. M. Bartz, A. R. Bentley, L. F. Bielak, M. Chong, A. Y. Chu, D. Berry, R. Dorajoo, N. D. Dueker, E. Kasbohm, B. Feenstra, M. F. Feitosa, C. Gieger, M. Graff, L. M. Hall, T. Haller, F. P. Hartwig, D. A. Hillis, V. Huikari, N. Heard-Costa, C. Holzapfel, A. U. Jackson, Å. Johansson, A. M. Jørgensen, M. A. Kaakinen, R. Karlsson, K. F. Kerr, B. Kim, C. M. Koolhaas, Z. Kutalik, V. Lagou, P. A. Lind, M. Lorentzon, L. P. Lyytikäinen, M. Mangino, C. Metzendorf, K. R. Monroe, A. Pacolet, L. Pérusse, R. Pool, R. C. Richmond, N. V. Rivera, S. Robiou-du-Pont, K. E. Schraut, C. A. Schulz, H. M. Stringham, T. Tanaka, A. Teumer, C. Turman, P. J. van der Most, M. Vanmunster, F. J. A. van Rooij, J. V. van Vliet-Ostaptchouk, X. Zhang, J. H. Zhao, W. Zhao, Z. Balkhiyarova, M. N. Balslev-Harder, S. E. Baumeister, J. Beilby, J. Blangero, D. I. Boomsma, S. Brage, P. S. Braund, J. A. Brody, M. Bruinenberg, U. Ekelund, C. T. Liu, J. W. Cole, F. S. Collins, L. A. Cupples, T. Esko, S. Enroth, J. D. Faul, L. Fernandez-Rhodes, A. E. Fohner, O. H. Franco, T.

E. Galesloot, S. D. Gordon, N. Grarup, C. A. Hartman, G. Heiss, J. Hui, T. Illig, R. Jago, A. James, P. K. Joshi, T. Jung, M. Kähönen, T. O. Kilpeläinen, W. P. Koh, I. Kolcic, P. P. Kraft, J. Kuusisto, L. J. Launer, A. Li, A. Linneberg, J. Luan, P. M. Vidal, S. E. Medland, Y. Milaneschi, A. Moscati, B. Musk, C. P. Nelson, I. M. Nolte, N. L. Pedersen, A. Peters, P. A. Peyser, C. Power, O. T. Raitakari, M. Reedik, A. P. Reiner, P. M. Ridker, I. Rudan, K. Ryan, M. A. Sarzynski, L. J. Scott, R. A. Scott, S. Sidney, K. Siggeirsdottir, A. V. Smith, J. A. Smith, E. Sonestedt, M. Strøm, E. S. Tai, K. K. Teo, B. Thorand, A. Tönjes, A. Tremblay, A. G. Uitterlinden, J. Vangipurapu, N. van Schoor, U. Völker, G. Willemsen, K. Williams, Q. Wong, H. Xu, K. L. Young, J. M. Yuan, M. C. Zillikens, A. B. Zonderman, A. Ameur, S. Bandinelli, J. C. Bis, M. Boehnke, C. Bouchard, D. I. Chasman, G. D. Smith, E. J. C. de Geus, L. Deldicque, M. Dörr, M. K. Evans, L. Ferrucci, M. Fornage, C. Fox, T. Garland, V. Gudnason, U. Gyllensten, T. Hansen, C. Hayward, B. L. Horta, E. Hyppönen, M. R. Jarvelin, W. C. Johnson, S. L. R. Kardia, L. A. Kiemeney, M. Laakso, C. Langenberg, T. Lehtimäki, L. Le Marchand, B. Z. Alizadeh, H. M. Boezen, L. Franke, M. Swertz, C. Wijmenga, P. van der Harst, G. Navis, M. Rots, B. H. R. Wolffenbuttel, P. K. E. Magnusson, N. G. Martin, M. Melbye, A. Metspalu, D. Meyre, K. E. North, C. Ohlsson, A. J. Oldehinkel, M. Orho-Melander, G. Pare, T. Park, O. Pedersen, B. W. J. H. Penninx, T. H. Pers, O. Polasek, I. Prokopenko, C. N. Rotimi, N. J. Samani, X. Sim, H. Snieder, T. I. A. Sørensen, T. D. Spector, N. J. Timpson, R. M. van Dam, N. van der Velde, C. M. van Duijn, P. Vollenweider, H. Völzke, T. Voortman, G. Waeber, N. J. Wareham, D. R. Weir, H. E. Wichmann, J. F. Wilson, A. L. Hevener, A. Krook, J. R. Zierath, M. A. I. Thomis, R. J. F. Loos, M. den Hoed, Nat. Genet. 2022, 54, 1332.

[27] E. Eising, N. Mirza-schreiber, E. L. De Zeeuw, C. A. Wang, D. T. Truong, A. G. Allegrini, C. Yang, K. M. Price, V. M. Rajagopal, Proc. Natl. Acad. Sci. 2022, 119, e2202764119.

[28] J. J. Lee, R. Wedow, A. Okbay, E. Kong, O. Maghzian, M. Zacher, T. A. Nguyen-Viet, P. Bowers, J. Sidorenko, R. Karlsson Linnér, M. A. Fontana, T. Kundu, C. Lee, H. Li, R. Li, R. Royer, P. N. Timshel, R. K. Walters, E. A. Willoughby, L. Yengo, M. Agee, B. Alipanahi, A. Auton, R. K. Bell, K. Bryc, S. L. Elson, P. Fontanillas, D. A. Hinds, J. C. McCreight, K. E. Huber, N. K. Litterman, M. H. McIntyre, J. L. Mountain, E. S. Noblin, C. A. M. Northover, S. J. Pitts, J. F. Sathirapongsasuti, O. V. Sazonova, J. F. Shelton, S. Shringarpure, C. Tian, V. Vacic, C. H. Wilson, J. P. Beauchamp, T. H. Pers, C. A. Rietveld, P. Turley, G. B. Chen, V. Emilsson, S. F. W. Meddens,

S. Oskarsson, J. K. Pickrell, K. Thom, P. Timshel, R. de Vlaming, A. Abdellaoui, T. S. Ahluwalia, J. Bacelis, C. Baumbach, G. Bjornsdottir, J. H. Brandsma, M. P. Concas, J. Derringer, N. A. Furlotte, T. E. Galesloot, G. Girotto, R. Gupta, L. M. Hall, S. E. Harris, E. Hofer, M. Horikoshi, J. E. Huffman, K. Kaasik, I. P. Kalafati, R. Karlsson, A. Kong, J. Lahti, S. J. van der Lee, C. de Leeuw, P. A. Lind, K. O. Lindgren, T. Liu, M. Mangino, J. Marten, E. Mihailov, M. B. Miller, P. J. van der Most, C. Oldmeadow, A. Payton, N. Pervjakova, W. J. Peyrot, Y. Qian, O. Raitakari, R. Rueedi, E. Salvi, B. Schmidt, K. E. Schraut, J. Shi, A. V. Smith, R. A. Poot, B. St Pourcain, A. Teumer, G. Thorleifsson, N. Verweij, D. Vuckovic, J. Wellmann, H. J. Westra, J. Yang, W. Zhao, Z. Zhu, B. Z. Alizadeh, N. Amin, A. Bakshi, S. E. Baumeister, G. Biino, K. Bønnelykke, P. A. Boyle, H. Campbell, F. P. Cappuccio, G. Davies, J. E. De Neve, P. Deloukas, I. Demuth, J. Ding, P. Eibich, L. Eisele, N. Eklund, D. M. Evans, J. D. Faul, M. F. Feitosa, A. J. Forstner, I. Gandin, B. Gunnarsson, B. V. Halldórsson, T. B. Harris, A. C. Heath, L. J. Hocking, E. G. Holliday, G. Homuth, M. A. Horan, J. J. Hottenga, P. L. de Jager, P. K. Joshi, A. Jugessur, M. A. Kaakinen, M. Kähönen, S. Kanoni, L. Keltigangas-Järvinen, L. A. L. M. Kiemeney, I. Kolcic, S. Koskinen, A. T. Kraja, M. Kroh, Z. Kutalik, A. Latvala, L. J. Launer, M. P. Lebreton, D. F. Levinson, P. Lichtenstein, P. Lichtner, D. C. M. Liewald, L. L. C. S. A. Loukola, P. A. Madden, R. Mägi, T. Mäki-Opas, R. E. Marioni, P. Marques-Vidal, G. A. Meddens, G. McMahon, C. Meisinger, T. Meitinger, Y. Milaneschi, L. Milani, G. W. Montgomery, R. Myhre, C. P. Nelson, D. R. Nyholt, W. E. R. Ollier, A. Palotie, L. Paternoster, N. L. Pedersen, K. E. Petrovic, D. J. Porteous, K. Räikkönen, S. M. Ring, A. Robino, O. Rostapshova, I. Rudan, A. Rustichini, V. Salomaa, A. R. Sanders, A. P. Sarin, H. Schmidt, R. J. Scott, B. H. Smith, J. A. Smith, J. A. Staessen, E. Steinhagen-Thiessen, K. Strauch, A. Terracciano, M. D. Tobin, S. Ulivi, S. Vaccargiu, L. Quaye, F. J. A. van Rooij, C. Venturini, A. A. E. Vinkhuyzen, U. Völker, H. Völzke, J. M. Vonk, D. Vozzi, J. Waage, E. B. Ware, G. Willemsen, J. R. Attia, D. A. Bennett, K. Berger, L. Bertram, H. Bisgaard, D. I. Boomsma, I. B. Borecki, U. Bültmann, C. F. Chabris, F. Cucca, D. Cusi, I. J. Deary, G. V. Dedoussis, C. M. van Duijn, J. G. Eriksson, B. Franke, L. Franke, P. Gasparini, P. V. Gejman, C. Gieger, H. J. Grabe, J. Gratten, P. J. F. Groenen, V. Gudnason, P. van der Harst, C. Hayward, W. Hoffmann, E. Hyppönen, W. G. Iacono, B. Jacobsson, M. R. Järvelin, K. H. Jöckel, J. Kaprio, S. L. R. Kardia, T. Lehtimäki, S. F. Lehrer, P. K. E. Magnusson, N. G. Martin, M. McGue, A. Metspalu, N. Pendleton, B. W. J. H. Penninx, M. Perola, N. Pirastu, M. Pirastu, O. Polasek, D. Posthuma, C. Power, M. A. Province, N. J. Samani, D. Schlessinger, R.

Schmidt, T. I. A. Sørensen, T. D. Spector, K. Stefansson, U. Thorsteinsdottir, A. R. Thurik, N. J. Timpson, H. Tiemeier, J. Y. Tung, A. G. Uitterlinden, V. Vitart, P. Vollenweider, D. R. Weir, J. F. Wilson, A. F. Wright, D. C. Conley, R. F. Krueger, G. D. Smith, A. Hofman, D. I. Laibson, S. E. Medland, M. N. Meyer, J. Yang, M. Johannesson, P. M. Visscher, T. Esko, P. D. Koellinger, D. Cesarini, D. J. Benjamin, M. Alver, Y. Bao, D. W. Clark, F. R. Day, K. E. Kemper, A. Kleinman, C. Langenberg, J. W. Trampush, S. S. Verma, Y. Wu, M. Lam, J. H. Zhao, Z. Zheng, J. D. Boardman, J. Freese, K. M. Harris, P. Herd, M. Kumari, T. Lencz, J. Luan, A. K. Malhotra, K. K. Ong, J. R. B. Perry, M. D. Ritchie, M. C. Smart, N. J. Wareham, M. R. Robinson, C. Watson, P. Turley, Nat. Genet. 2018, 50, 1112.

[29] S. Burgess, A. Butterworth, S. G. Thompson, Genet. Epidemiol. 2013, 37, 658.

[30] J. Bowden, G. D. Smith, S. Burgess, Int. J. Epidemiol. 2015, 44, 512.

[31] M. Verbanck, C. Y. Chen, B. Neale, R. Do, Nat. Genet. 2018, 50, 693.

[32] S. Burgess, N. M. Davies, S. G. Thompson, Genet. Epidemiol. 2016, 40, 597.

[33] T. J. Vanderweele, Annu. Rev. Public Heal. 2016, 37, 17.

[34] D. Tofighi, D. P. Mackinnon, Behav. Res. Methods 2011, 43, 692.

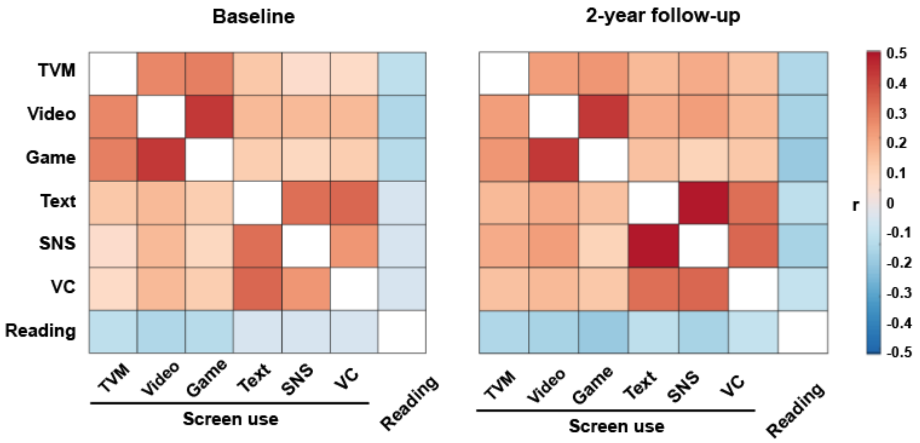


**Figure S1**. The Pearson correlation among all exposure variables at baseline (left) and 2-year follow-up (right). Abbreviation: TVM = watching television shows/ movies; Video: watching videos; Game = playing video games; Text = texting on a cell phone, tablet, and so on; SNS = visiting social networking sites; VC = video chatting.

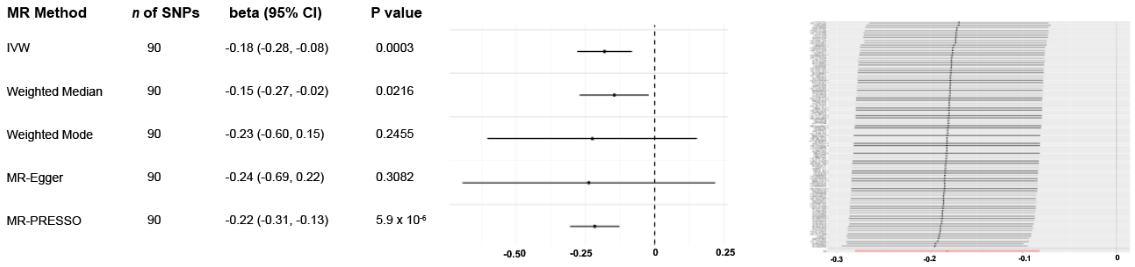


**Figure S2**. MR estimation of the association between screen use and reading ability. Estimates showed the effect size (95% Cis) for the change in reading ability due to leisure screen use. The right panel shows the leave-one-out plot of the instrumental SNPs.

| MR Method | *n* of SNPs | beta (95% CI) | P value |
|---|---|---|---|
| IVW | 87 | -0.19 (-0.26, -0.12) | $1.34 \times 10^{-8}$ |
| Weighted Median | 87 | -0.10 (-0.15, -0.05) | $7.20 \times 10^{-5}$ |
| Weighted Mode | 87 | -0.07 (-0.18, 0.05) | 0.2599 |
| MR-Egger | 87 | -0.21 (-0.51, 0.09) | 0.1665 |
| MR-PRESSO | 87 | -0.15 (-0.19, -0.10) | $1.72 \times 10^{-8}$ |

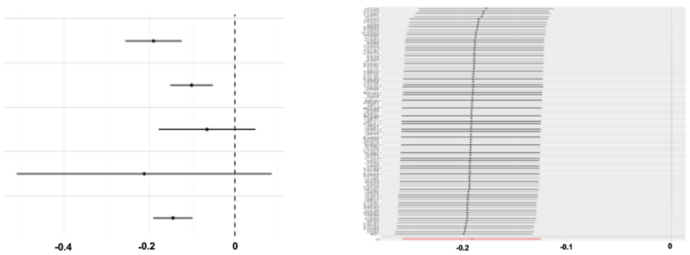


**Figure S3**. MR estimation of the association between screen use and cognitive performance. Estimates showed the effect size (95% Cis) for the change in cognitive performance due to leisure screen use. The right panel shows the leave-one-out plot of the instrumental SNPs.

| MR Method | *n* of SNPs | beta (95% CI) | P value |
|---|---|---|---|
| IVW | 109 | -0.20 (-0.28, -0.04) | $5.26 \times 10^{-9}$ |
| Weighted Median | 109 | -0.18 (-0.25, -0.12) | $9.06 \times 10^{-8}$ |
| Weighted Mode | 109 | -0.10 (-0.33, 0.13) | 0.3981 |
| MR-Egger | 109 | -0.20 (-0.52, 0.12) | 0.2228 |
| MR-PRESSO | 109 | -0.24 (-0.30, -0.19) | $1.96 \times 10^{-14}$ |

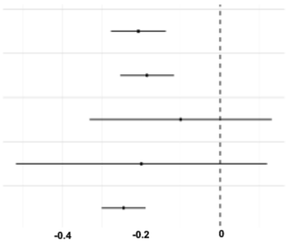


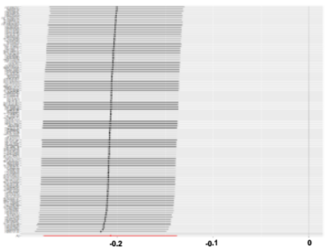


**Figure S4**. Reverser MR estimation of the association between screen use and cognitive performance. Estimates showed the effect size (95% Cis) for the change of screen use time due to cognitive performance. The right panel shows the leave-one-out plot of the instrumental SNPs.
